# A narrative review on the role of IL-1β, IL-8, and the endometrial matrix in heavy menstrual bleeding pathogenesis

**DOI:** 10.1097/MS9.0000000000003820

**Published:** 2025-09-01

**Authors:** Emmanuel Ifeanyi Obeagu

**Affiliations:** Department of Biomedical and Laboratory Science, Africa University, Mutare, Zimbabwe

**Keywords:** endometrial matrix, heavy menstrual bleeding, IL-1β, IL-8, inflammation

## Abstract

Heavy menstrual bleeding (HMB) is a common gynecological condition characterized by excessive menstrual blood loss, often leading to anemia and reduced quality of life. Inflammation plays a crucial role in its pathogenesis, with interleukin-1 beta (IL-1β) and interleukin-8 (IL-8) emerging as key mediators in endometrial dysfunction. IL-1β amplifies inflammatory responses, stimulates matrix metalloproteinases (MMPs), and promotes extracellular matrix (ECM) degradation, leading to increased tissue breakdown and vascular instability. Concurrently, IL-8 recruits neutrophils and drives abnormal angiogenesis, contributing to excessive and prolonged bleeding. The interaction between these cytokines and the ECM disrupts endometrial homeostasis, exacerbating tissue shedding and vascular fragility. IL-1β-induced MMP activation degrades ECM components such as collagen and laminin, weakening the structural integrity of the endometrial lining. Meanwhile, IL-8-mediated neutrophil infiltration and reactive oxygen species release further promote tissue damage. Additionally, IL-8 enhances endothelial cell proliferation, leading to the formation of fragile, easily ruptured blood vessels that exacerbate HMB symptoms.

## Introduction

Heavy menstrual bleeding (HMB) is one of the most common gynecological disorders, affecting up to 30% of reproductive-age women. It is defined as excessive menstrual blood loss exceeding 80 mL per cycle and is often associated with anemia, fatigue, and a significant decline in quality of life. The underlying causes of HMB are diverse, including hormonal imbalances, coagulation disorders, uterine structural abnormalities, and chronic inflammation. Emerging evidence suggests that inflammatory cytokines, particularly interleukin-1 beta (IL-1β) and interleukin-8 (IL-8), play a crucial role in the dysregulation of endometrial function, leading to excessive menstrual bleeding^[1–5]^. Cytokines are key mediators of immune responses and tissue remodeling in the endometrium. The menstrual cycle is a dynamic process involving cycles of endometrial proliferation, differentiation, and shedding, all of which are tightly regulated by cytokines and growth factors. IL-1β and IL-8, in particular, have been implicated in endometrial inflammation, extracellular matrix (ECM) remodeling, and vascular changes. Dysregulation of these cytokines disrupts the delicate balance required for normal menstruation, leading to increased vascular permeability, excessive leukocyte infiltration, and abnormal ECM breakdown – all of which contribute to HMB^[6–9]^.

**HIGHLIGHTS**

- IL-1β triggers inflammation, promoting matrix degradation and endometrial instability.
- IL-8 attracts neutrophils, amplifying local inflammatory responses in the endometrium.
- Endometrial matrix breakdown is accelerated by cytokine-induced MMP activation.
- Synergistic action of IL-1β and IL-8 disrupts vascular integrity, increasing bleeding.
- Cytokine-matrix interplay underlies chronic endometrial dysfunction in heavy menstrual bleeding.

IL-1β is a potent pro-inflammatory cytokine that amplifies immune responses by stimulating the release of additional inflammatory mediators. It activates matrix metalloproteinases (MMPs), which degrade ECM components such as collagen and fibronectin, thereby destabilizing endometrial tissue. Additionally, IL-1β increases vascular permeability and promotes angiogenesis, leading to fragile and leaky blood vessels that contribute to excessive menstrual bleeding. Studies have shown that IL-1β levels are elevated in the endometrial tissue of women with HMB, suggesting a strong association between IL-1β-driven inflammation and abnormal menstrual bleeding[10]. IL-8, a chemokine primarily involved in neutrophil recruitment and activation, also plays a pivotal role in endometrial function. Under normal conditions, IL-8 facilitates leukocyte infiltration to aid in tissue breakdown and immune defense. However, excessive IL-8 production leads to increased neutrophil activation, release of proteolytic enzymes, and oxidative stress, all of which contribute to exaggerated ECM degradation and endometrial instability. Additionally, IL-8 promotes endothelial cell proliferation and abnormal angiogenesis, leading to structurally weak blood vessels that rupture easily, further exacerbating menstrual blood loss^[11,12]^. The ECM serves as a structural scaffold for endometrial tissue and regulates cellular interactions critical for tissue stability and regeneration. The IL-1β/IL-8 axis significantly influences ECM homeostasis by altering the balance between MMPs and their tissue inhibitors of metalloproteinases (TIMPs). An overactive MMP response, triggered by IL-1β, results in excessive ECM degradation, while IL-8-mediated neutrophil infiltration further amplifies the inflammatory cascade. This disruption in ECM integrity weakens the endometrial lining and contributes to prolonged and heavy bleeding[13].

HMB is a prevalent gynecological concern affecting women across all reproductive age groups, with significant implications for quality of life and health system burden. According to the *Global Incidence Study 2023*, HMB affects an estimated 18–25% of menstruating women worldwide, with higher prevalence reported in low-resource settings where access to diagnostic and therapeutic services remains limited. The WHO estimates that HMB accounts for nearly one-third of all gynecologic outpatient visits and contributes substantially to iron-deficiency anemia in women of reproductive age. Despite its clinical significance, the biological underpinnings of HMB remain incompletely understood, particularly the role of inflammatory cytokines in its diverse etiologic subtypes. HMB is a heterogeneous condition encompassing structural (e.g., fibroids), ovulatory, coagulopathic, iatrogenic, and idiopathic causes. However, current research has not adequately delineated cytokine expression patterns, including IL-1β and IL-8, across these subtypes. For instance, it remains unclear whether women with ovulatory HMB exhibit distinct inflammatory signatures compared to those with coagulopathic or iatrogenic forms. This knowledge gap limits the development of targeted diagnostics and therapies and highlights a critical direction for future research: defining inflammatory phenotypes specific to each HMB subtype to enable more personalized and effective interventions^[14–18]^.

### Aim

The aim of this review is to explore the pathophysiological mechanisms underlying HMB, with a focus on the role of inflammatory cytokines such as IL-1β and IL-8 in endometrial dysfunction, matrix degradation, and vascular instability.

### Review methods

The review methodology employed involved a comprehensive and systematic examination of both primary research and clinical studies related to HMB, specifically focusing on the inflammatory cytokines IL-1β and IL-8, their roles in endometrial dysfunction, and the therapeutic interventions targeting these pathways. The review process consisted of several key stages: literature search, selection of relevant studies, data synthesis, and analysis of treatment approaches.

## Literature search

A thorough literature search was conducted using multiple databases, including PubMed, Google Scholar, Scopus, and Embase. The search terms used included “heavy menstrual bleeding,” “IL-1β,” “IL-8,” “endometrial inflammation,” “matrix degradation,” “cytokines in HMB,” and “therapeutic strategies for HMB.” Both basic science studies focusing on the molecular mechanisms and clinical trials evaluating therapeutic interventions were considered. Relevant articles were selected based on their relevance to the cytokine pathways involved in HMB and their potential clinical applications.

## Selection criteria

The selection criteria for inclusion focused on studies that investigated the role of IL-1β and IL-8 in the pathogenesis of HMB, as well as those that assessed the effectiveness of various medical, surgical, and novel therapies targeting these pathways. Articles that explored the underlying molecular mechanisms of endometrial dysfunction, matrix degradation, and vascular instability were prioritized. Studies examining the outcomes of cytokine-targeted therapies, anti-inflammatory treatments, and alternative approaches were also included. Exclusion criteria included studies that lacked robust methodological rigor, non-English language articles, and those with insufficient data on cytokine-related mechanisms in HMB.

## Limitations

While this review highlights important insights into the potential roles of IL-1β, IL-8, and the endometrial matrix in the pathogenesis of HMB, several limitations must be acknowledged. First, there is a notable paucity of direct clinical trial data evaluating IL-1β and IL-8-targeted therapies specifically in women with HMB. Much of the therapeutic discussion in this review is therefore extrapolated from preclinical models or studies of related inflammatory and gynecologic conditions, which may not fully reflect the unique pathophysiology of HMB. Second, the existing body of literature may be subject to publication bias, with a tendency to report positive findings more frequently than negative or inconclusive data. This bias may skew the perceived strength of associations between inflammatory markers and HMB severity. Third, HMB is a highly heterogeneous condition, encompassing structural, hormonal, coagulopathic, and idiopathic causes. The degree to which inflammatory pathways, such as IL-1β and IL-8 signaling, contribute across these subtypes remains incompletely understood. This heterogeneity limits the generalizability of pathophysiological mechanisms and therapeutic extrapolations across all women with HMB. Finally, variations in study design, diagnostic criteria, and sample characteristics across the reviewed studies further complicate direct comparisons and synthesis. Future studies with standardized methodologies and subgroup-specific analyses are needed to clarify the role of inflammation across the spectrum of HMB etiologies.

## IL-1β in endometrial inflammation and matrix degradation in heavy menstrual bleeding

HMB is a prevalent gynecological condition characterized by excessive and prolonged menstrual blood loss, often resulting in anemia and a diminished quality of life. A growing body of evidence implicates inflammatory processes in the pathogenesis of HMB, with IL-1β playing a pivotal role. IL-1β, a key pro-inflammatory cytokine, is primarily secreted by activated macrophages, neutrophils, and endometrial stromal cells in response to tissue injury and immune activation. While IL-1β is essential for normal endometrial shedding during menstruation, its dysregulated expression in HMB leads to excessive inflammation, ECM degradation, and vascular instability, culminating in prolonged and heavy bleeding^[19,20]^. IL-1β mediates its effects by binding to the interleukin-1 receptor (IL-1R), activating intracellular signaling pathways such as nuclear factor kappa B and mitogen-activated protein kinases. These signaling cascades drive the production of inflammatory mediators, including prostaglandins and leukotrienes, which enhance vascular permeability and increase immune cell infiltration into the endometrium. Studies have shown that women with HMB exhibit significantly elevated IL-1β levels in their endometrial tissue, correlating with heightened inflammatory responses and dysregulated menstrual shedding[21]. A critical mechanism by which IL-1β contributes to HMB is through the upregulation of MMPs, particularly MMP-2 and MMP-9. MMPs are responsible for degrading ECM components such as collagen, fibronectin, and laminin, facilitating endometrial breakdown during menstruation. However, in HMB, excessive IL-1β stimulation leads to uncontrolled MMP activation, resulting in premature and excessive ECM degradation. This weakens the endometrial lining, making it more susceptible to abnormal shedding and excessive menstrual blood loss. Furthermore, IL-1β downregulates TIMPs, further exacerbating ECM degradation and disrupting the delicate balance necessary for normal endometrial repair[22].

Beyond ECM breakdown, IL-1β influences angiogenesis and vascular function, both of which are crucial for endometrial homeostasis. It stimulates the production of vascular endothelial growth factor (VEGF), promoting the formation of new blood vessels. However, in HMB, IL-1β-driven angiogenesis results in the development of structurally weak, hyperpermeable blood vessels that rupture easily, contributing to excessive bleeding. The combination of fragile vasculature, increased vascular permeability, and impaired endothelial repair further aggravates the pathophysiology of HMB[23]. The inflammatory effects of IL-1β are further amplified by its ability to recruit and activate immune cells, particularly neutrophils and macrophages. These immune cells release additional pro-inflammatory cytokines, reactive oxygen species (ROS), and proteolytic enzymes, perpetuating a cycle of inflammation and tissue damage. Neutrophil extracellular traps, composed of DNA and antimicrobial proteins, have been identified in the endometrial tissue of women with HMB, further linking excessive immune activation to menstrual dysfunction[16]. Given its central role in endometrial inflammation and ECM degradation, IL-1β represents a promising therapeutic target for managing HMB. Potential interventions include IL-1R antagonists (e.g., Anakinra), anti-inflammatory agents that suppress IL-1β production, and MMP inhibitors that prevent excessive ECM breakdown. Additionally, lifestyle modifications and dietary interventions that regulate inflammatory pathways may provide adjunctive benefits in reducing HMB severity (Tables 1 and 2)[24].

**Table 1:** Mechanistic roles of IL-1β, IL-8, and ECM disruption in HMB pathogenesis

Factor	Mechanistic role in HMB pathogenesis	Key evidence sources
IL-1β	- Triggers endometrial inflammation- Upregulates matrix metalloproteinases (MMPs), especially MMP-9- Promotes ECM degradation and leukocyte infiltration	Mei *et al*, 2015; Aziz *et al*, 2020
IL-8 (CXCL8)	- Acts as a potent neutrophil chemoattractant- Induces aberrant angiogenesis and endothelial cell activation- Associated with increased menstrual blood loss via vascular instability	Donnez *et al*, 2019; Salamonsen *et al*, 2009
ECM Disruption	- Loss of collagen integrity (e.g., types I and IV)- MMP-driven degradation leads to fragile vasculature- Reduces endometrial tensile strength, facilitating excessive bleeding	Salamonsen *et al*, 2009; Hannan *et al*, 2012

**Table 2:** Biological effects and interactions of IL-1β, IL-8, and ECM remodeling in HMB

Molecule	Interaction with MMPs	Impact on angiogenesis	Effect on vascular permeability
IL-1β	Induces MMP-2 and MMP-9 expression in stromal cells	Promotes VEGF expression and new vessel formation	Increases endothelial leakiness and vascular fragility
IL-8 (CXCL8)	Indirectly upregulates MMP-9 via neutrophil activation	Enhances aberrant neovascularization	Disrupts endothelial junctions, increasing permeability
ECM (collagen, laminin, fibronectin)	Substrates for MMP cleavage during menstruation	Disrupted ECM supports disorganized angiogenesis	Loss of structural support increases vessel rupture risk

## IL-8 and neutrophil recruitment in endometrial dysfunction in heavy menstrual bleeding

HMB is a debilitating gynecological condition characterized by excessive and prolonged menstrual blood loss. While hormonal imbalances and structural abnormalities are commonly implicated, increasing evidence suggests that immune dysregulation plays a significant role in its pathogenesis. IL-8, a potent chemokine, is a key regulator of neutrophil recruitment and activation within the endometrium. Although IL-8 is essential for normal endometrial shedding and repair, its excessive expression in HMB contributes to chronic inflammation, uncontrolled ECM degradation, and abnormal vascular remodeling, all of which exacerbate excessive bleeding[25]. IL-8 is primarily produced by endometrial epithelial cells, stromal cells, and immune cells in response to inflammatory stimuli such as IL-1β and tumor necrosis factor-alpha (TNF-α). Acting through its receptors, CXCR1 and CXCR2, IL-8 orchestrates the migration and activation of neutrophils into the endometrial tissue. In women with HMB, elevated IL-8 levels have been correlated with increased neutrophil infiltration, leading to prolonged inflammatory responses and excessive tissue breakdown. The sustained presence of neutrophils amplifies local inflammation by releasing additional pro-inflammatory cytokines, proteolytic enzymes, and ROS, which further disrupt endometrial homeostasis[26].

Neutrophils play a crucial role in ECM degradation, a process essential for normal endometrial shedding. Upon activation by IL-8, neutrophils release MMPs, particularly MMP-9, which degrade structural components of the ECM, including collagen and fibronectin. While controlled ECM degradation is necessary for normal menstruation, excessive neutrophil-derived MMP activity in HMB leads to premature and extensive ECM breakdown, weakening the endometrial structure and contributing to increased menstrual blood loss. Furthermore, neutrophil elastase, another proteolytic enzyme, exacerbates ECM degradation and interferes with proper tissue repair, prolonging menstrual bleeding[27]. Beyond its effects on ECM remodeling, IL-8 also influences angiogenesis, the process of new blood vessel formation. In the normal endometrium, IL-8-mediated angiogenesis ensures adequate tissue perfusion and repair after menstruation. However, in HMB, IL-8-driven angiogenesis results in the formation of fragile, dysfunctional blood vessels that are prone to rupture. This leads to increased vascular permeability and impaired hemostasis, further contributing to excessive menstrual bleeding. Additionally, neutrophils recruited by IL-8 enhance endothelial cell activation and vascular destabilization, worsening blood loss during menstruation[28]. IL-8’s role in HMB extends beyond neutrophil recruitment and vascular remodeling; it also sustains a chronic inflammatory environment in the endometrium. Acting in synergy with IL-1β and other cytokines, IL-8 perpetuates immune cell infiltration, disrupts the balance between MMPs and their inhibitors, and impairs normal endometrial repair mechanisms. This chronic inflammatory state prevents the resolution of menstruation, leading to recurrent episodes of excessive bleeding[29]. Given its pivotal role in neutrophil recruitment, ECM degradation, and vascular dysfunction, IL-8 represents a potential therapeutic target for managing HMB. Inhibiting IL-8 signaling through CXCR1/2 antagonists, anti-inflammatory agents, or monoclonal antibodies may help regulate neutrophil infiltration, reduce excessive ECM breakdown, and restore vascular stability. Additionally, MMP inhibitors that counteract IL-8-driven proteolysis could provide a novel approach to reducing menstrual blood loss in affected women[30].

## The role of the endometrial matrix in heavy menstrual bleeding

The endometrial ECM plays a crucial role in maintaining the structural integrity and functional stability of the endometrium. It provides a scaffold for cellular interactions, regulates immune responses, and facilitates the cyclic processes of endometrial regeneration, shedding, and repair. However, in HMB, dysregulation of ECM components leads to excessive tissue breakdown, impaired repair mechanisms, and abnormal vascular remodeling, all of which contribute to prolonged and excessive blood loss. Understanding the complex interplay between ECM degradation, inflammatory mediators, and vascular instability is essential for unraveling the pathophysiology of HMB and identifying potential therapeutic targets^[31,32]^. The ECM of the endometrium is composed of various structural proteins, including collagen, fibronectin, laminin, and proteoglycans, which provide mechanical support and regulate cellular adhesion and signaling. These components undergo controlled degradation during menstruation, allowing for the cyclical shedding and renewal of the endometrial lining. However, in HMB, there is an imbalance between MMPs and their natural inhibitors, tissue inhibitors of TIMPs. MMPs, particularly MMP-2 and MMP-9, are responsible for ECM degradation, facilitating endometrial shedding. In women with HMB, excessive MMP activity leads to premature and excessive ECM breakdown, weakening the endometrial structure and increasing menstrual blood loss^[33,34]^.

Inflammatory cytokines, particularly IL-1β and IL-8, play a pivotal role in modulating ECM dynamics. IL-1β stimulates MMP production, amplifying ECM degradation and perpetuating a cycle of excessive tissue breakdown. IL-8 further exacerbates this process by recruiting neutrophils, which release proteolytic enzymes such as neutrophil elastase and MMP-9. These enzymes not only degrade ECM proteins but also impair endometrial repair, prolonging bleeding episodes. The sustained inflammatory response in HMB disrupts the delicate balance required for efficient ECM turnover, leading to structural instability and persistent menstrual dysfunction[3]. Beyond its role in structural integrity, the ECM is crucial for vascular stability and angiogenesis. The endometrial microvasculature is embedded within the ECM, and its integrity is dependent on proper ECM composition and remodeling. In HMB, excessive ECM degradation results in the formation of fragile, leaky blood vessels that are prone to rupture, exacerbating menstrual blood loss. Additionally, VEGF, a key regulator of angiogenesis, is upregulated in response to ECM breakdown. While VEGF-mediated angiogenesis is essential for endometrial repair, its dysregulated expression in HMB contributes to the development of abnormal, hyperpermeable blood vessels, further worsening blood loss^[35,36]^. Another critical aspect of ECM dysfunction in HMB is its impact on endometrial repair mechanisms. Under normal conditions, the ECM serves as a reservoir for growth factors such as transforming growth factor-beta and fibroblast growth factors, which promote tissue regeneration. However, excessive ECM degradation depletes these factors, impairing the ability of the endometrium to undergo efficient repair after menstruation. This results in prolonged bleeding and increased susceptibility to recurrent episodes of HMB. Furthermore, alterations in the composition of ECM glycoproteins, such as fibronectin and laminin, may disrupt cellular adhesion and migration, further delaying endometrial regeneration^[37,38]^.

## Synergistic inflammatory pathways and extracellular matrix remodeling in HMB

Emerging evidence suggests that the inflammatory microenvironment of the endometrium in HMB is not solely driven by individual cytokines, but rather by synergistic interactions between key mediators such as IL-1β and TNF-α. These cytokines act in concert to amplify the local immune response, particularly during the perimenstrual phase. IL-1β and TNF-α synergistically enhance neutrophil recruitment via upregulation of chemokines such as IL-8 (CXCL8), leading to increased ROS generation, protease release, and amplified production of downstream cytokines like IL-6 and GM-CSF. This creates a positive feedback loop of inflammation that can exacerbate tissue breakdown and vascular permeability. Such cooperative signaling has been shown to upregulate MMPs, further driving ECM degradation and compromising endometrial vascular integrity^[39,40]^.

In the context of ECM remodeling, recent studies have drawn attention to specific alterations in collagen composition in women with HMB. Notably, a disproportionate reduction in collagen types I and III expression has been observed in the perivascular regions of the endometrium. Collagen I, known for its tensile strength, and collagen III, which contributes to vascular compliance and repair, are essential for maintaining microvascular stability. The depletion or disorganization of these structural proteins leads to increased vessel fragility, impaired tissue remodeling, and excessive menstrual blood loss. Furthermore, degraded collagen fragments may themselves act as damage-associated molecular patterns, perpetuating inflammatory signaling and MMP activation^[41,42]^.

## Therapeutic perspectives in heavy menstrual bleeding

HMB is a common condition that significantly impacts the quality of life for many women, leading to physical, emotional, and social distress. The management of HMB is multifaceted, often requiring a combination of medical, surgical, and lifestyle interventions to address the underlying causes and alleviate symptoms. Although several treatment options are available, selecting the most appropriate approach depends on the severity of bleeding, underlying etiologies, and the individual’s reproductive goals. The therapeutic landscape for HMB is evolving, with new treatments focused on targeting the pathophysiological mechanisms that contribute to excessive bleeding, such as inflammation, endometrial dysfunction, and vascular abnormalities[43].

## Medical treatments

The first-line medical treatment for HMB often involves hormonal therapies, including oral contraceptives, progestins, and intrauterine devices (IUDs) containing levonorgestrel. These therapies work by thinning the endometrial lining, reducing menstrual blood loss, and providing symptomatic relief. Progestins and hormonal IUDs are particularly effective for women with dysfunctional uterine bleeding and those with underlying conditions like fibroids or adenomyosis. Additionally, non-hormonal agents, such as tranexamic acid and nonsteroidal anti-inflammatory drugs (NSAIDs), are commonly used to reduce bleeding and alleviate pain by inhibiting fibrinolysis and reducing inflammation. Tranexamic acid, in particular, has been shown to decrease menstrual blood loss by stabilizing the fibrin clot, while NSAIDs help manage pain and reduce prostaglandin production, which is linked to excessive bleeding[39]. For women who do not respond to conventional hormonal therapy or those with contraindications to these treatments, oral medications like desmopressin and antifibrinolytics may be beneficial, particularly for those with bleeding disorders such as von Willebrand disease or platelet dysfunction[44]. In addition, medications that target the inflammatory pathways involved in endometrial dysfunction, such as corticosteroids or anti-cytokine agents, offer potential for managing bleeding in patients with chronic inflammatory conditions like endometriosis or adenomyosis. More recently, biologic therapies that target specific cytokines like IL-1β and IL-8, which are implicated in the inflammatory processes underlying HMB, have been explored as promising options[45].

## Surgical treatments

For women with persistent or severe HMB that does not respond to medical therapies, surgical options may be necessary. One of the most common surgical interventions is endometrial ablation, which involves the destruction of the endometrial lining to reduce or stop bleeding. This procedure is effective for women who do not wish to preserve fertility, as it can result in permanent cessation of menstruation. Another surgical approach is myomectomy, which is recommended for women with uterine fibroids that are contributing to HMB. Myomectomy involves the removal of fibroids while preserving the uterus and may be an option for women desiring future fertility. Hysterectomy, the complete removal of the uterus, is considered a last resort for women with refractory HMB, particularly when other treatments have failed, or if there are significant structural abnormalities[36]. Emerging surgical techniques, such as hysteroscopic surgery, allow for the removal or destruction of abnormal tissue in a minimally invasive manner. These procedures, including the resection of fibroids, polyps, or adenomyosis, provide alternatives to more invasive surgeries like hysterectomy and can lead to a reduction in menstrual bleeding while preserving reproductive function. Hysteroscopic myomectomy and polypectomy have been associated with reduced bleeding, improved fertility outcomes, and shorter recovery times compared to traditional open surgery[46].

## Emerging and novel therapeutic approaches

In addition to conventional therapies, innovative treatments are being developed to address the underlying pathophysiological mechanisms of HMB. These include targeting the molecular and inflammatory pathways that drive excessive bleeding. For example, therapies that focus on the inhibition of MMPs, which play a crucial role in endometrial matrix degradation, have shown promise in regulating abnormal tissue breakdown and reducing excessive menstrual loss. Similarly, targeting cytokines like IL-1β and IL-8, which mediate inflammatory responses and neutrophil recruitment, offers the potential to reduce endometrial inflammation and matrix degradation associated with HMB[38]. Gene therapies, including those aimed at modulating angiogenesis and regulating vascular stability, are also being explored. For instance, the inhibition of VEGF, which is upregulated in response to endometrial tissue breakdown, could prevent the formation of abnormal blood vessels and reduce the fragility of the endometrial vasculature, thereby minimizing excessive bleeding. The development of selective estrogen receptor modulators and other targeted therapies that focus on the regulation of estrogen activity may also offer new treatment options, particularly for women with conditions like endometriosis or adenomyosis, where estrogen plays a significant role in disease progression[47].

## Lifestyle and non-pharmacological interventions

In addition to medical and surgical treatments, lifestyle modifications can play an important role in managing HMB. Maintaining a healthy weight, engaging in regular exercise, and managing stress are all factors that can influence hormonal balance and reduce menstrual bleeding. Nutritional interventions, such as increasing the intake of iron-rich foods or iron supplementation, can help mitigate the anemia associated with HMB. For some women, acupuncture and other complementary therapies may provide symptom relief, although more research is needed to establish their efficacy in the treatment of HMB[44].

## Psychological and emotional support

Given the significant impact that HMB can have on a woman’s physical and mental well-being, psychological and emotional support should be integrated into treatment plans. Cognitive-behavioral therapy and counseling can help women manage the emotional burden associated with chronic menstrual bleeding, particularly for those who experience anxiety, depression, or diminished quality of life due to the condition. Providing women with access to support groups or counseling services can also help normalize their experience and reduce the stigma surrounding menstrual health[48].

## Medical treatments and emerging therapeutics targeting IL-1β, IL-8, and the endometrial matrix

Current medical management of HMB largely focuses on hormonal modulation, antifibrinolytics (e.g., tranexamic acid), and in selected cases, agents such as desmopressin for those with bleeding disorders. However, a growing body of evidence implicates dysregulated inflammation – particularly involving IL-1β, interleukin-8 (IL-8/CXCL8), and ECM degradation – in the pathogenesis of HMB. This has prompted exploration of novel therapeutics that target these molecular pathways^[49]^.

## IL-1β inhibition: Anakinra and related agents

IL-1β is a potent proinflammatory cytokine upregulated during menstruation and has been shown to induce MMPs, disrupt endometrial integrity, and promote vascular leakage. Anakinra, a recombinant IL-1R antagonist approved for rheumatoid arthritis and other autoinflammatory conditions, has demonstrated favorable modulation of MMP expression and vascular permeability in various preclinical models. While no clinical trials have yet evaluated Anakinra specifically for HMB, rodent models of endometriosis and uterine inflammation show that IL-1β blockade reduces leukocyte infiltration, angiogenesis, and tissue destruction – hallmarks relevant to HMB pathophysiology (1,2). Moreover, *ex vivo* studies on human endometrial stromal cells treated with IL-1β inhibitors have reported downregulation of MMP-9 and VEGF, suggesting potential for therapeutic stabilization of the endometrial matrix (3). Given these findings, repurposing IL-1β inhibitors warrants investigation in women with inflammation-driven HMB, particularly those unresponsive to hormonal therapy[44].

## Anti-CXCL8/CXCR2 therapies

IL-8 (CXCL8), a key neutrophil chemoattractant and angiogenic mediator, has been associated with excessive leukocyte recruitment and vascular fragility in the endometrium of women with HMB. Experimental therapies targeting CXCL8 or its receptor CXCR2 have shown efficacy in reducing neutrophil-driven inflammation and pathological angiogenesis in gynecologic and autoimmune disease models. For instance, CXCR2 antagonists (e.g., reparixin) have demonstrated anti-inflammatory and anti-angiogenic effects in endometriosis models and in early-phase clinical trials for other inflammatory conditions (4,5). Although direct data in HMB are lacking, these mechanistic parallels support the hypothesis that targeting the CXCL8/CXCR2 axis could mitigate inflammatory endometrial degradation and abnormal bleeding[46].

## Conclusion

This review highlights the pivotal roles of IL-1β, IL-8, and ECM disruption in the complex pathogenesis of HMB, emphasizing how inflammatory cascades and matrix degradation synergistically compromise endometrial vascular integrity. While current therapeutic options largely focus on hormonal modulation and symptomatic management, emerging evidence underscores the potential of targeting inflammatory pathways to improve outcomes. A practical recommendation arising from this synthesis is the implementation of early screening for inflammatory markers, such as menstrual fluid IL-1β and IL-8 assays, particularly in women with unexplained HMB or those exhibiting poor response to conventional hormonal therapies. This approach may be especially valuable for patients at heightened risk of coagulopathies or immune dysregulation, enabling personalized treatment strategies. Future prospective studies are essential to validate the clinical utility of such biomarkers and to explore targeted anti-inflammatory interventions in HMB management.

## Data Availability

Not applicable, as this is a Narrative Review.

## Research registration unique identifying number (UIN)

Not applicable, as this is a Narrative Review.
